# Design and Evaluation of a Just-in-Time Adaptive Intervention (JITAI) to Reduce Sedentary Behavior at Work: Experimental Study

**DOI:** 10.2196/34309

**Published:** 2022-01-26

**Authors:** Tasnim Ismail, Dena Al Thani

**Affiliations:** 1 Division of Information and Computing Technology College of Science and Engineering Hamad Bin Khalifa University Doha Qatar

**Keywords:** sedentary behavior, persuasive technology, behavior change, physical activity, adaptive intervention

## Abstract

**Background:**

Employees in sedentary occupations tend to spend prolonged hours physically inactive. Physical inactivity is a main factor in the increase in the risks of a wide range of chronic diseases, including obesity, diabetes, hypertension, and heart disease. This has drawn researchers’ attention to investigate methods of increasing the level of activity of employees during working hours and in their daily lifestyle.

**Objective:**

The objective of this paper is to investigate the effectiveness of using personalized messages that include user information, user goals, daily routine, and the surrounding environment to increase the level of activity among employees. In this study, we hypothesize that sending context-aware motivational messages to workers in sedentary occupations after sitting for 40 minutes can break sedentary behavior and increase daily active time compared to static reminder messages.

**Methods:**

A 66-day between-group study using a mixed methods design approach was conducted with employees who are located in Qatar and spend most of their working day sedentary. The 58 participants used 2 different interventions: The control group (n=29, 50%) used a mobile app that only sends a static message after prolonged sitting (MotiFit Lite), and the intervention group (n=29, 50%) used a mobile app that sends context-aware personalized messages to promote physical activity (PA; MotiFit). Both apps log the received messages, the step count before and after the messages are sent, and the user response to the messages to obtain an idea of the impact of the messages. The study received approval from the Qatar Biomedical Research Institute’s institutional review board (IRB application #2019-10-037).

**Results:**

The questionnaires showed satisfaction of the designed apps’ subjective quality and perceived impact. The quantitative analysis showed a high level of engagement in the intervention group compared to the control group (*P*<.001). The results support the original hypothesis that using context-aware motivational messages can increase PA at work compared to static messages (*P*<.001). However, the analysis showed no significant impact of the message type on the overall activity level during the day (*P*=.06).

**Conclusions:**

Context-aware motivational messages motivate employees to increase their PA in the workplace. However, future research will further develop the analysis to investigate the impact on increasing the overall activity level during the day.

## Introduction

An inactive lifestyle is a main controllable cause of metabolic syndrome (MetS), along with obesity and insulin resistance [1]. MetS is defined according to the International Diabetes Federation (IDF) as the group of risk factors that raise the likelihood of getting health complications, such as heart disease, diabetes, and stroke [2]. These risk factors can be in the form of traits, conditions, or habits. PA benefits health in many ways, including the prevention of obesity, hypertension, heart disease, stroke, type II diabetes mellitus, and hypercholesterolemia [3]. Moreover, some studies have proved that an active work style improves creativity, self-esteem, mental health, and stress tolerance, as well as work performance and productivity [4,5]. Studies have also shown that frequent rest breaks are successful in reducing self-reported discomfort in the upper limb muscles by up to 35%, while improving work productivity and speed [6].

Mobile health coaching approaches have many benefits over traditional face-to-face methods because they are more accessible, scalable, cost-effective, and time-location independent. They can also be personalized to meet the user’s needs and preferences. This personalization makes mobile apps getting used by more people generally and makes mobile apps popular for motivating PAs particularly [7]. Moreover, mobile apps use phone sensors, such as accelerometers, gyroscopes, and the Global Positioning System (GPS). The use of an accelerometer embedded into mobiles for measuring PAs has been evaluated, and the results have shown accuracy and reliability in measuring and quantifying PAs in both laboratory and real-life settings [8].

According to the just-in-time support, following an intervention-determined scheme rather than a participant-determined scheme provides the right type of support at the right time [9]. Multiple components are used to construct the foundation to design these interventions: decision points, intervention options, tailoring variables, and decision rules. A decision point in just-in-time adaptive intervention (JITAI) is the time at which an intervention decision is made [10]. An intervention option is a set of possible actions that would be used at a given decision point for different types, methods, and amounts of support, delivered through a particular medium. Tailoring variables are information concerning an individual that is used to decide which intervention to offer and at what time. Tailoring variables in JITAI can be attained using ongoing assessment, passive assessment, or both. Finally, the decision rules in JITAI systematically link the intervention options and the tailoring variables to operationalize the adaption by specifying which intervention option to provide, for whom, and when [11].

Mobile apps in health care are equipped with supportive functionalities to make them more persuasive toward behavioral change. For example, some apps make use of the sensors integrated into smartphones and handle the interaction through a persuasive mobile app [12]. In a pilot study, researchers presented an activity logger mobile app (BEN’FIT) that aims to motivate PA by suggesting equipment-free exercises that can be done in the home or work environment. In another study, a mobile app (SitCoach) was investigated for the effectiveness of using reminders after prolonged sitting time to reduce sedentary behavior at work [13]. Another study used gamification to design an app based on self-determination theory that records user steps, then converts them into points, and allows the users to compete with each other, hence motivating them to walk more [14].

Other studies have combined fitness tracking with context awareness to come up with persuasive suggestions to improve well-being. One mobile app, for example, detects active and inactive behaviors and uses time, location, weather, and personal information to achieve its motivational target [15]. Another aims to encourage users to walk more by using their location and weather information and then an outdoor detour map to recommend walks. The results of evaluation among 8 participants showed user satisfaction with the app, especially logging and visualization of PAs. However, some participants highlighted that sedentary behavior alerts are frequent. Additionally, the study revealed that the participants would follow the recommendations only when the suggestion is easy. Participants also suggested to use a mobile app for step count acquisition to avoid prolonged phone-on-table periods.

In this study, we investigate the effectiveness of using context-aware and personalized messages in breaking sedentary behavior. We propose a mobile app, MotiFit, that is designed to increase the level of PA among employees in sedentary occupations by encouraging them to walk more. The approach followed is to send personalized motivational messages to workers after prolonged sitting to remind them to walk. The proposed personalization takes advantage of user goals, location, weather information, and daily routine to employ context awareness in the generated motivational messages. The study evaluates the effectiveness of the designed messages to advocate positive behavior change and encourage workers to walk more, hence maintaining a healthier lifestyle. To prove the study hypothesis, we evaluated the MotiFit app for 66 days with a mixed methods design. We hypothesized that sending context-aware motivational messages to workers in sedentary occupations after prolonged sitting can break sedentary behavior and increase daily active time.

## Methods

### Overview of the Approach Followed

The study investigated the effectiveness of personalized, context-aware motivational messages in breaking the sedentary behavior of employees in sedentary occupations and increasing their daily activity level compared to static reminders. First, participants were given a prestudy online questionnaire for collecting their demographic information (Multimedia Appendix 1) and the International Physical Activity Questionnaire (IPAQ) to determine the level of PA before the study, hence distributing the participants with different PA levels equally into 2 groups [16]. Then, the participants were given the mobile app that automatically logs their step count. Participants in the control group received static reminders, while participants in the intervention group received context-aware motivational messages, and when the participants received them, they were asked to rate the messages in terms of correctness and relatability.

After the duration of the study elapsed, the participants were given the user version of the Mobile Application Rating Scale (uMARS) questionnaire to measure the usability of the app [17]. All the participants were invited to a semistructured interview to obtain information about the context of use. The interview included some questions about the overall app experience in terms of usability, functions, and features. In addition, some questions were directed toward the challenges that the participants encountered and suggestions for enhancements.

The study followed the between-group design, where the participant is only exposed to 1 condition so that they do not learn from different task conditions. Since the participants only need to complete tasks under 1 condition, the time it takes each participant to complete the study is much shorter than in a within-group design. As a result, exhaustion and frustration can be effectively controlled [18].

**Figure 1:**
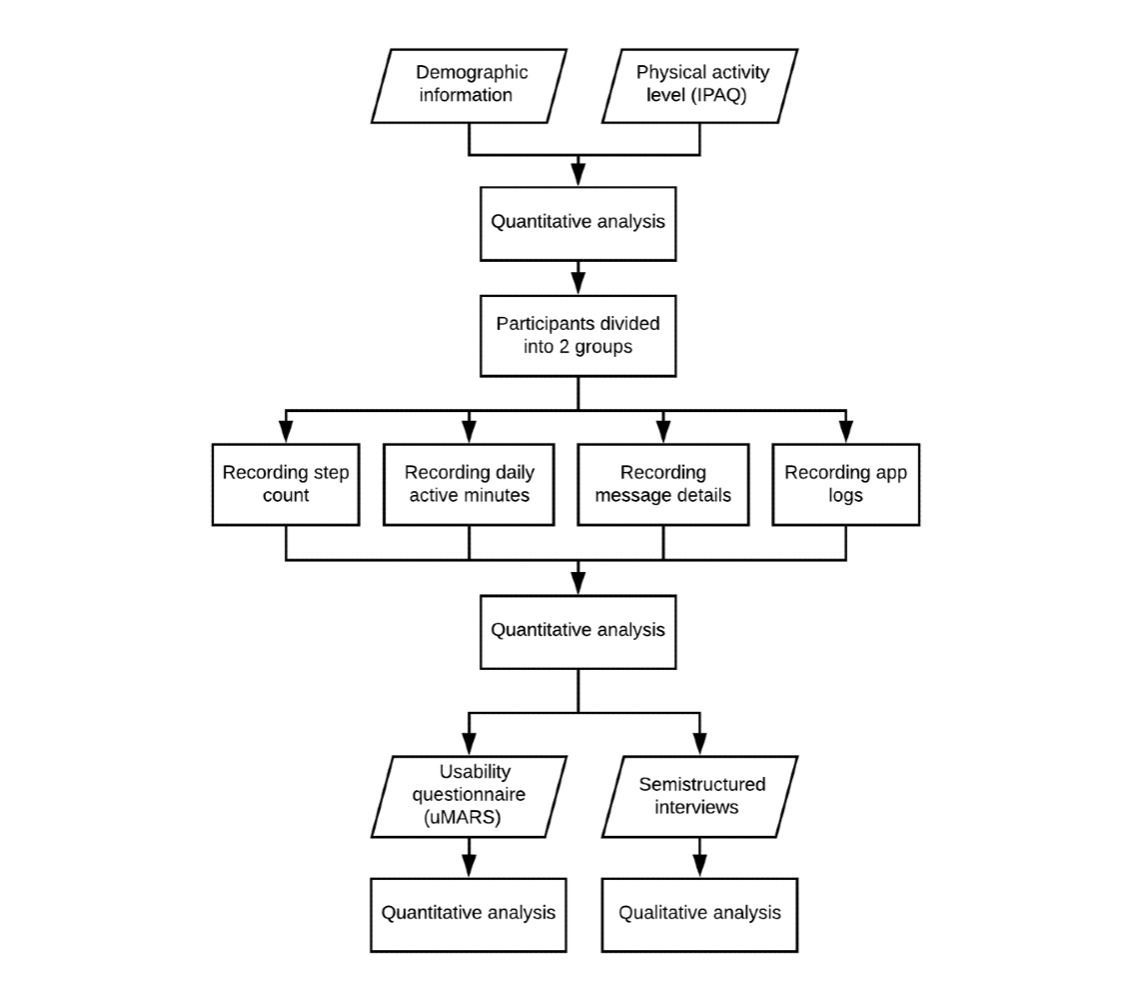
Study procedure. IPAQ: International Physical Activity Questionnaire; uMARS: user version of the Mobile Application Rating Scale.

In this study, the thematic analysis approach was followed to examine the participants’ perspectives to generate unanticipated insights. This method involves an iterative and reflective process to simplify the text into codes that focus on the important characteristics of the data, then sort them into themes that bring meaning to the data [19]. Figure 1 summarizes the overall approach followed in the study.

We received approval from the Qatar Biomedical Research Institute’s institutional review board for all users who participated in this study (IRB application #2019- 10-037). Participation was entirely voluntary, and participants were sent an approved informed consent form, which contained all the details about the study. All participants were assured of their data confidentiality and security and informed that they have the right to withdraw from the study without justifications or penalties.

### Study Hypotheses

In this study, we explored the impact of context-aware motivational messages against static reminders on the PA level following a mixed methods research approach applied in a field study. A causal-comparative quantitative approach was followed to examine the effect of the independent variable on the dependent variable within a cause-effect relationship. The dependent variable was the inactivity intervals. The independent variables were the possible causes of the change in the dependent variable. The investigated causes, or what were known as the independent variable, were the context-aware motivational messages against static reminders.

Experimental studies should be based on a predefined hypothesis, not driven by data [18]. Hence, it is critical to identify the study hypotheses at an early stage as given next.

#### Hypothesis 1

Sending context-aware motivational messages to workers in sedentary occupations after sitting for 40 minutes can break sedentary behavior compared to static reminder messages.

The static reminder message is a message that does not change its content, for example, “Hey, you've been sitting for long. How about you take a short walk?”

Sedentary behavior is defined as jobs that require sitting for most of the working time, including computer professions [20].

#### Hypothesis 2

Sending context-aware motivational messages to workers in sedentary occupations after sitting for 40 minutes can increase daily active time compared to static reminder messages.

Active time increase is measured by the time the user spends moving during the day.

### Recruitment

In total, 58 participants, all sedentary workers in different sectors in Qatar, were recruited following the snowballing sampling method. Participants were selected on the basis of the following inclusion criteria: having a predominantly sedentary job and working at least 6 hours a day, 5 days a week; age between 23 and 39 years; having the ability to walk, since the study was based on accelerometer data; owning an Android smartphone; and having a level of understanding of the English language, enough to understand the app and answer the questionnaires. The participants were divided into a control group and an intervention group. The intervention group consisted of 29 participants (50%: 21 [72%] within the age range of 23-30 years, 19 [66%] females). The control group consisted of 29 participants (50%: 21 [72%] within the age range of 23-30 years, 17 [59%] females).

### Study Duration

The participants were asked to use the mobile app for 66 days in their daily routine. The duration choice was based on an empirical study that investigated the time required for a behavioral change to happen. It was conducted over the range of 18-254 days, and it found that on average, it takes 66 days for a repeated behavior to reach its maximum level of automaticity [21].

### App Design

**Figure 2:**
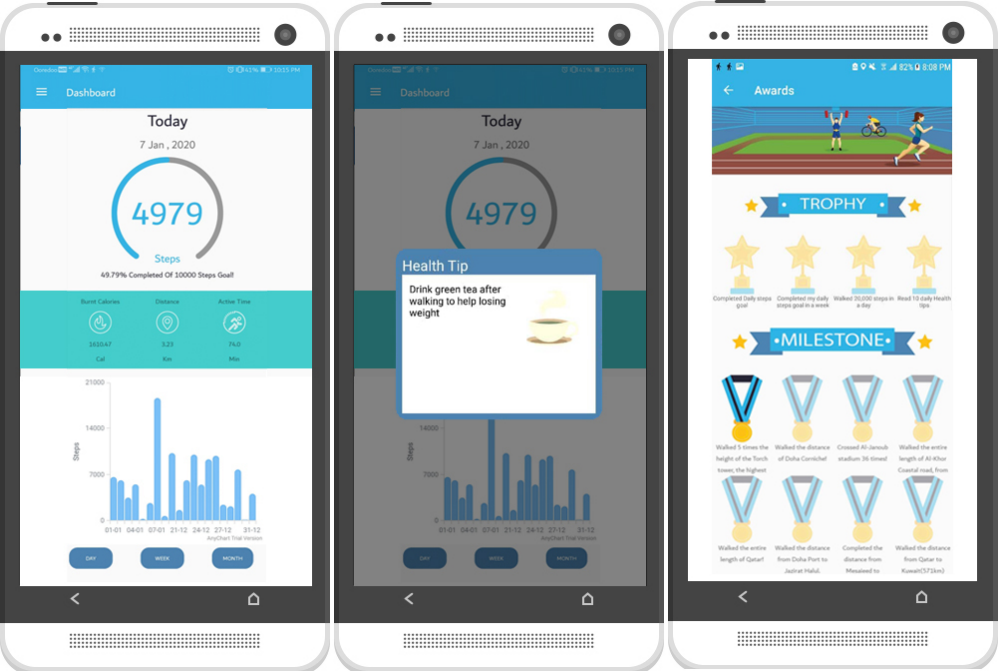
MotiFit app screens: (a) main dashboard, (b) motivational message, and (c) achievements.

The MotiFit app was designed to be easy to use in terms of the methods to input user information and output the processed data in a visual and textual format. The app is designed not to consume time such that data are automatically recorded and user interruption is minimal. The app does not require a lot of effort physically; for example, the given tasks are easy for various fitness levels. The messages are designed considering the social environment and culture, so they are familiar, and they do not go out of the norm culturally. All these elements are considered together to ensure that the target audience is able to effectively use the app toward achieving the behavioral change goal. Based on this reasoning, the app was created as a persuasive technology app that focuses on positive social, behavioral change. Figure 2 gives an overview of the app prototype.

### Motivational Message Design

To form new habits, we considered 2 key concepts: focusing on 1 habit at a time and connecting the new behavior to already existing activities [21]. Hence, MotiFit focuses only on the walking habit, and the motivational messages are designed to persuade walking through context awareness, considering the surrounding environment and the user’s routine. Experts recommended engaging the new behavior with daily activities, such as eating and sleeping [22]; hence, MotiFit checks for lunch breaks and the end of working hours during weekdays at the time of sending messages to suggest activities that can fit during the particular time frame.

#### Rest Duration Choice

A study concluded that microbreaks every 40 minutes help reduce discomfort in the shoulders, wrists, and backs of the participants, with no evidence of microbreak effect on productivity reduction at work [23]. For this study, the app was set to send motivational messages every 40 minutes, and the duration of the breaks was estimated as 1-3 minutes. This duration is equivalent to at least 67 steps to break sedentary behavior [24].

#### Location

The location information retrieved is used to find nearby locations of different destinations, such as restaurants, gyms, parks, or malls, depending on the situation when the message is sent. Some locations are chosen to be reached by walking, and for this, locations within 500 m are retrieved. Other locations are chosen to be reached by driving; for the lunch break, locations within 1.5 km are retrieved, while for after working hours, locations within 6 km are retrieved.

#### Weather Information

Weather information helps identify the nature of the activity that the user can do, whether indoors or outdoors. The weather information is fetched using the Open Weather Application Programming Interface (API), which returns 3 parameters: temperature, humidity level, and weather forecast. Based on these parameters, the classification of suitable and unsuitable weather for outdoor PAs is performed. Information shows the classification of weather information, as performed by Gupta and Sood [25]. Since the study was conducted in Qatar during the winter season, we adjusted the suitable temperature range, as shown in Table 1, to 13-35C.

**Table 1:** Classification of weather information.

Parameter	Suitable level	Unsuitable level
Temperature, C	13-35	>35
Humidity level, %	<90	>90
Forecast	Clear skies, sunny, cloudy	Windy, rainy, thunderstorm, hailstorm

#### Time Information

The decision point in JITAI is the time at which an intervention decision is made. In this study, we used prespecified time intervals based on the duration of inactivity [10]. The messages are designed to be triggered 30 minutes before lunch break if the user is inactive in order to allow the user to make lunch plans accordingly. The motivational messages that are triggered before the end of the working day are sent 1 hour before so that receivers get enough time to arrange after-work plans. The same thing applies to messages that are triggered before the end of the day such that users get enough time to finish their daily step goals, if possible.

#### Motivational Message Structure

**Figure 3:**
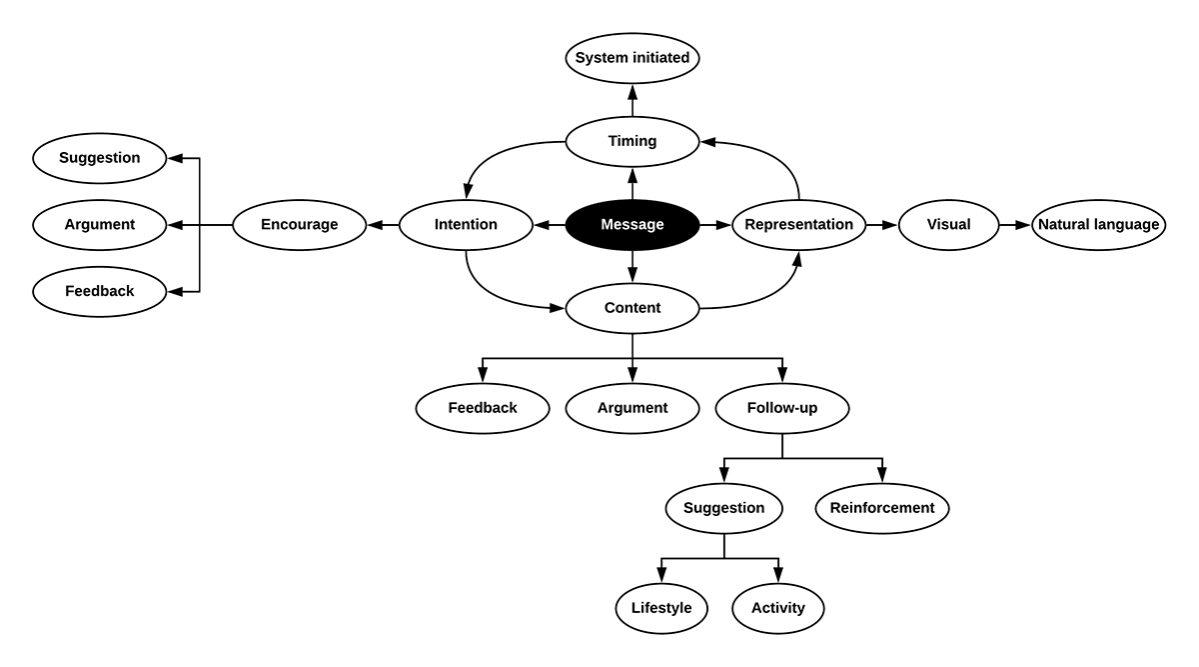
Modified model of motivational message generation.

The motivational message model follows a modified version of Akker et al’s [26] model, as shown in Figure 3. The modified model is a subset of the Akker model, which starts with a system-initiated trigger based on the time with an intention to encourage walking behavior through suggestions, arguments, and feedback. Then, the content of the message is identified, whether it is feedback, an argument, or a follow-up on progress. Finally, the content of the message is represented in an adaptive textual format and sent to the user as a mobile app notification.

The structure of the motivational message consists of 2 parts: a heading and a message body. The heading is a short, catchy phrase, and the body of the message has the actual content. The body of the message uses slot filling to adapt dynamic content based on the fetched data when the message is sent, as shown in Multimedia Appendix 2.

#### Motivational Message Algorithm

The choice of the messages is made following a simple decision-making flowchart, as shown in Multimedia Appendix 3. A total of 260 unique motivational messages were generated based on the flowchart. Although the messages are divided into 15 categories, the messages within the same category consist of 2 main types: messages targeting self-efficacy and framed messages [27]. Messages targeting self-efficacy are those messages that tackle an individual’s belief in their capacity to perform some behavior. Message framing, however, can be gain-framed, focusing on the positive outcomes, or loss-framed, focusing on the costs and losses [28]. Our analysis further investigated the impact of the different types of messages.

## Results

### Sample Characteristics

The first set of study results were obtained for the control group, which used the lite version of the app that always sends the same static message (MotiFit Lite). The second set of results was obtained for the intervention group, which used the full version of the app that sends personalized motivational messages (MotiFit).

Table 2 describes the demographics of the sample. A total of 58 participants were included in the study. The participants were divided into a control group (n=29, 50%) and an intervention group (n=29, 50%). The participants of the 2 groups were mostly female (36/58, 62%), and they fell in the 23-30-year age group. Most of the participants were bachelor’s degree holders (33/58, 57%), and their current occupation was under the office work category (30/58, 52%). With regard to the fitness level, the participants had different body mass index (BMI) levels. from underweight to obese.

The IPAQ classifies the participants’ PA level into 3 categories: low, moderate, and high. The classification is based on the participants’ weekly PAs based on their energy requirements defined in metabolic equivalents (METs). METs are multiples of the resting metabolic rate, and a MET minute (MET-min) is computed by multiplying the MET score of the activity by the minutes performed [29]:




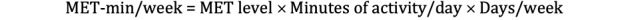




where MET level=3.3 METs for walking, 4.0 METs for moderate-intensity PA, and 8.0 METS for vigorous-intensity PA.

**Figure 4:**
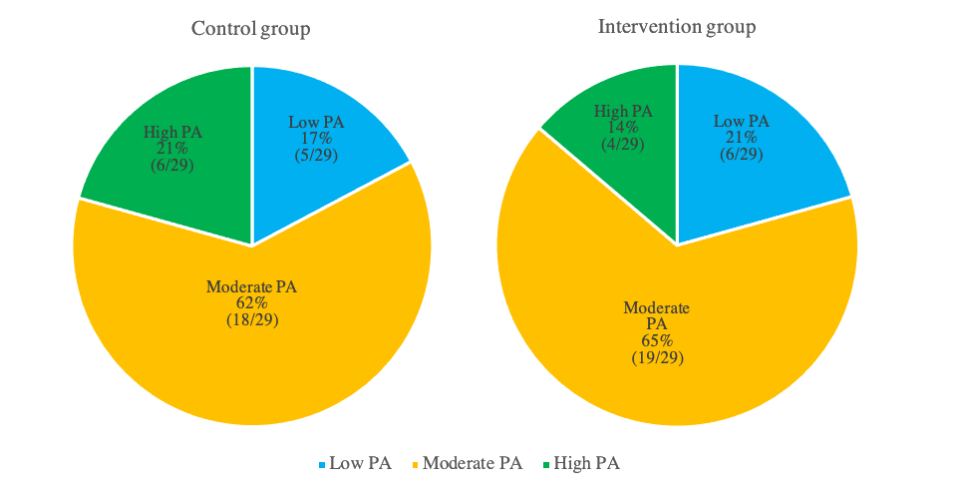
Participants' distribution over PA categories. PA: physical activity.

According to the scoring guide of the IPAQ, a person is classified in the low-active group if they do less than 600 MET-min/week, in the moderate-active group if they do more than 600 MET-min/week and less than 3000 MET-min/week, or in the high-active group if they do more than 3000 MET-min/week [29]. The IPAQ classification was used mainly to distribute participants with different PA levels into control and intervention groups, as shown in Figure 4. This classification was used to eliminate bias from the recruitment process.

**Table 2:** Participants’ demographic information (N=58).

Characteristics		Control group (n=29, 50%)	Intervention group (n=29, 50%)
**Gender, n (%)**			
	Female	19 (66)	17 (59)
	Male	10 (34)	12 (41)
**Age (years), n (%)**			
	23-30	21 (72)	21 (72)
	31-39	8 (28)	8 (28)
**BMI^a^ assessment, n (%)**			
	Underweight	1 (3)	0 (0)
	Normal	12 (41)	17 (59)
	Overweight	9 (31)	11 (38)
	Obese	7 (24)	1 (3)
**Education, n (%)**			
	High school/diploma	8 (28)	0 (0)
	Bachelor’s degree	14 (48)	19 (66)
	Master’s degree	6 (21)	8 (28)
	Doctorate	1 (3)	2 (7)
**Work status, n (%)**			
	Academics	8 (28)	6 (21)
	Engineering	0 (0)	2 (7)
	Health profession	1 (3)	1 (3)
	IT	3 (10)	2 (7)
	Office work	16 (55)	14 (48)
	Sales and services	1 (3)	4 (14)

^a^BMI: body mass index.

### User Engagement

The study took place over 66 days. However, not all participants completed the full duration. The full duration defined here is the duration for which each participant was enrolled in the study, from the starting date to the ending date of using MotiFit. The number of days for which the reminder messages were sent was defined based on the logged data. In addition, the logs showed the number of days for which the app was running, but no messages were sent to the participant. This could be due to an increase in the participant’s PA or a technical issue in the app.

**Figure 5:**
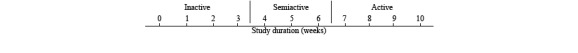
Participants' categorization based on their engagement in the study.

From the resulting data, we decided to follow the approach used by Al-Ansari et al [30] to classify the participants’ engagement into 3 categories: inactive, semiactive, and active. The participants who used the app for 3 weeks or less were considered inactive, those who used the app for 4-6 weeks were considered semiactive, and those who used the app for 7 weeks or more were considered active, as shown in Figure 5. The distribution of participants over the engagement categories was summarized for both control and intervention groups, as shown in Table 3.

We wanted to further investigate the relationship between user engagement and the gender within the intervention group. The results in Table 4 showed no statistically significant difference between genders, with a chi-square test result of *χ*^2^=1.5 (*P*=.47). We also investigated the relationship between user engagement and age within the intervention group. The results showed no statistically significant difference between the 2 age groups, with a chi-square test result of *χ*^2^=2.7 (*P*=.26).

We also used data obtained from Google Analytics to track the participants’ engagement on a daily basis based on the app’s screens. Considering a significance level of .05, the average time spent surfing the main dashboard was significantly higher in the intervention group, with a Mann-Whitney result of W=119 (*P*<.001). Since the main dashboard contains all the activity-level trackers, this can tell us that the intervention group participants were more interested in the activity-level details and comparison than the control group participants.

**Table 3:** Engagement levels of control and intervention groups (N=58).

Engagement level	Control group (n=29, 50%)	Intervention group (n=29, 50%)
Inactive, n (%)	11 (38)	1 (3)
Semiactive, n (%)	12 (41)	12 (41)
Active, n (%)	6 (21)	16 (55)

**Table 4:** Engagement levels in the intervention group between gender and age (n=29).

Characteristic		Inactive participants, n (%)	Semiactive participants, n (%)	Active participants, n (%)
**Gender**				
	Male	1 (3)	5 (17)	6 (21)
	Female	0 (0)	7 (24)	10 (35)
**Age (years)**				
	23-30	0 (0)	9 (31)	12 (41)
	31-39	1 (3)	3 (10)	4 (14)

### Logged Messages

**Figure 6:**
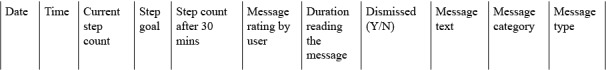
Data header attributes for intervention group messages.

The logged data header for the intervention group contained the following attributes shown in Figure 6.

For the control group, the same attributes were logged, except for the message type, category, and text, since the app always sends the same static message: “Hey, you've been sitting for long. How about you take a short walk?”

The message is marked with a flag that detects whether the message was dismissed from the notifications or whether it was viewed for less than 2 seconds. The choice of 2 seconds was made by estimating the read time of the shortest message using a read-o-meter tool [31]. Hence, we considered any message that was not dismissed by the participant but viewed for less than 2 seconds as dismissed.

From the messages that were not dismissed, the difference in steps within 30 minutes of sending the messages was calculated and then, a new attribute was generated to determine whether the messages broke the sedentary behavior: if the difference in the step count ≥67 steps, the user has broken their sedentary behavior; otherwise, they have not.

Table 5 shows the results after cleaning the data, which consist of 1125 records for the control group (MotiFit Lite) and 5228 records for the intervention group (MotiFit). The results were statistically significant, showing a chi-square test result of *χ*^2^=12.9 (*P*=.001), which indicates that the intervention group was using the app more actively compared to the control group.

To investigate the effectiveness in breaking inactivity, we combined the information in the message and the participant’s response to it in order to determine the impact of the message on breaking sedentary behavior. We recorded information about the type of message, an indication of whether the message was dismissed, and the difference in the step count before and after the message was received. Then, we performed the chi-square test to investigate the relationship between the message type and the breaking of inactivity, as shown in Table 5. The results were statistically significant (*χ*^2^= 410.1, *P*<.001). Hence, MotiFit has an impact on breaking inactivity.

We wanted to further study the impact of different categories of the messages used in the intervention group. The categories are gain-framed messages, loss-framed messages, and messages targeting self-efficacy. Of the messages that were not dismissed by the intervention group (n=5228), 3058 (58.49%) were categorized as gain-framed, 1479 (28.29%) were categorized as loss-framed, and 691 (13.22%) were categorized as messages targeting self-efficacy. The results for each category with an impact of the personalized context-aware messages on breaking inactivity are presented in Table 6.

The chi-square test was performed to investigate the relationship between different categories of messages with regard to breaking inactivity in the intervention group. The results were not statistically significant (*χ*^2^=4.2, *P*=.12). Hence, there is no direct impact of the category of the message on breaking inactivity.

**Table 5:** Messages’ (N=6353) impact on control and intervention groups.

Type of message	Messages that did not break inactivity (n=3348, 52.69%)	Messages that broke inactivity (n=3005, 47.31%)
Context-aware message, n (%)	2447 (73.09)	2781 (92.55)
Static message, n (%)	901 (26.91)	224 (7.45)

**Table 6:** Different message (N=5228) categories vs breaking inactivity in the intervention group.

Characteristic	Messages that did not break inactivity (n=2447, 46.81%)	Messages that broke inactivity (n=2781, 53.19%)
Gain-framed message, n (%)	1467 (60)	1591 (57.2)
Loss-framed message, n (%)	672 (27.5)	807 (29)
Messages targeting self-efficacy, n (%)	308 (13.8)	383 (13.8)

### Logged Active Minutes

This set consists of the active minutes per day, which includes all types of PAs done throughout the day. These data are automatically recorded through the app by the end of each day to determine the effectiveness of the app to increase daily active time. However, since not all participants completed the full duration of the study, we selected the 6 most engaged participants each from the control and intervention groups and tried to determine the maximum common period of using the app among these participants, which was found to be 39 days.

Next, we used the multivariant Mann-Kendall trend test to examine whether there was a monotonic trend (increasing or decreasing) of active time over the study period. The control group showed Z_MK_=0.42 (*P*=.67), while the intervention group showed Z_MK_=0.005 (*P*=.99). None of the 2 groups showed a significant monotonic trend in the daily active time. We also performed the Mann-Whitney *U* test to examine whether there was a difference in the daily active time between the intervention group and the control group [32]. The test showed that there was no significant difference (W=572, *P*=.06) in the daily active time between the 2 groups.

### Usability Results

The uMARS questionnaire was given to the participants to rate their experience with using the app in terms of the ease of use and the functionalities available. The questionnaire is divided into 3 sections: app quality, app subjective quality, and perceived impact. The rating is scored out of 5 based on the scoring criteria given by the questionnaire developers [17].

Generally, the uMARS questionnaire rates the usability of an app. Since both apps given to the control and intervention groups are almost the same, we did not expect significant differences in the scores. We performed the Mann-Whitney *U* test for the 3 sections (app quality, app subjective quality, and perceived impact) of the uMARS questionnaire. The analysis results did not show any significance for the questions in the app quality and app subjective quality sections. This was expected, given that the 2 apps are identical in terms of the user interface, user experience, and content.

Moreover, the results were significant for most of the questions in the perceived impact section. The overall perceived impact score of the control group was significantly higher than that of the intervention group (W=566, *P*=.02). The scores of the awareness question were significantly higher for the control group (W=435, *P*<.001). Similarly, for the attitude and help-seeking questions, the scores of the control group were significantly higher (W=556, *P*=.02 and W=588, *P*=.006, respectively). However, for knowledge, intention to change, and behavior change, there was no significant difference (W=500, *P*=.18; W=535, *P*=.06; and W=517, *P*=.10, respectively).

## Discussion

### Principal Findings

This study designed and evaluated a mobile health app for employees in sedentary occupations to promote walking and engage them in PA. The mobile app aims to remind and encourage employees to move after prolonged sitting. Integrating the findings from quantitative and qualitative analyses led to interferences, which described the impact of the intervention in a comprehensive way. Overall, the results emerged from the quantitative analysis were compatible with those from the qualitative analysis. We found that the app is engaging as most of the participants (16/29, 55%) were active throughout the study period. The quantitative analysis of Google Analytics data showed that participants were specifically interested in viewing the main dashboard, which contains all the activity-level trackers. Moreover, we found that the context-aware motivation messages sent by MotiFit encouraged users to take more breaks during their prolonged inactive time. However, the results did not show a significant difference in the daily active time. It can be argued that the number of records was small and that the duration was not enough to make an impact on the participants’ lifestyles, especially since many of the participants did not complete the full duration of the study. Moreover, many external factors could affect the results in a real-life setting, especially since the study was conducted in a period that included several holidays, which could affect the participants’ daily routine. The qualitative analysis of usability surveys showed no impact of MotiFit for knowledge, intention to change, and behavior change; however, the quantitative analysis revealed contrasting results with high engagement rates and more success rates to break sedentary behavior in the intervention group.

Considering the integrated findings from both analyses, we inferred that the mobile health design with JITAI is engaging and usable and can empower users to integrate PA in their daily routine. The results of this study can act as propositions that give researchers a solid starting point for future research on persuasion and behavioral change for health promotion.

### Limitations

The main limitations of the study were the small sample size, the participants' engagement to continue the study for the entire study period, and the type of the participant information collected.

A larger sample size could provide more insight into the effect of the intervention and increase the statistical test power. The timing of the study was unfortunate since the recruitment took place in December, which is a high season for holidays, academic breaks, and annual leaves for employees. Hence, recruitment and user engagement were challenging.

Another limitation was the type of user information fetched in the study, which affected the personalization of the messages. The user information was limited to gender, age, step goal, and timing information according to the IRB committee agreement. Therefore, the personalization of the messages was limited to our knowledge of the participants. If the acquired information is more inclusive such that it contains health status, calendar information, and hobbies, then the app can generate a bigger variety of yet precise messages.

### Conclusion

This study investigated the potential of developing a mobile health intervention to encourage people to become more active during their working hours. The intervention was designed specifically for workers who spend most of their day at work sitting, yet it can be used by anyone. Previous studies have investigated behavioral change through personalized notification messages. However, this study additionally considered the participant’s personal information, step goal, daily routine, and the surrounding environment. For this investigation, a mobile app called MotiFit was developed and evaluated to gain insights into the effectiveness of personalized electronic health interventions to break inactivity.

The results proved that context-aware motivational messages can effectively break sedentary behavior compared to static reminders. However, the results showed no monotonic trend in the daily active time over the study period among the most engaged participants. In addition, the results did not show a statistical significance between the message category (gain-framed, loss-framed, targeting self-efficacy) and breaking sedentary behavior.

Several ways of improving the study results could be followed by obtaining more insights into the target population characteristics and increasing the amount of data acquired. Another approach is to increase the sample size and the duration of the study, to make the messages more personalized, and to enhance the mobile app interface. Future work can include integrating social aspects such that users can share their achievements on their social networks, compete with each other, and track other users’ performance, which could increase user engagement and prolong the app usage period. In addition, the motivational messages if-else algorithm can further be enhanced to use artificial intelligence to provide more personalized and accurate messages.

## Appendix

Multimedia Appendix 1 Participants demographic information questionnaire.

Multimedia Appendix 2 Dynamic context attributes in motivational messages.

Multimedia Appendix 3 Motivational messages decision-making flowchart.

## Abbreviations

API: Application Programming Interface
BMI: body mass index
GPS: Global Positioning System
IPAQ: International Physical Activity Questionnaire
IRB: institutional review board
JITAI: just-in-time adaptive intervention
MET: metabolic equivalent
MetS: Metabolic Syndrome
PA: physical activity
uMARS: user version of the Mobile Application Rating Scale
