# Supplementary figures and images for: Design and Evaluation of a Just-in-Time Adaptive Intervention (JITAI) to Reduce Sedentary Behavior at Work: Experimental Study
Source: JMIR Form Res. 2022 Jan 26;6(1):e34309. doi: 10.2196/34309 (PMC8943689; doi:10.2196/34309)

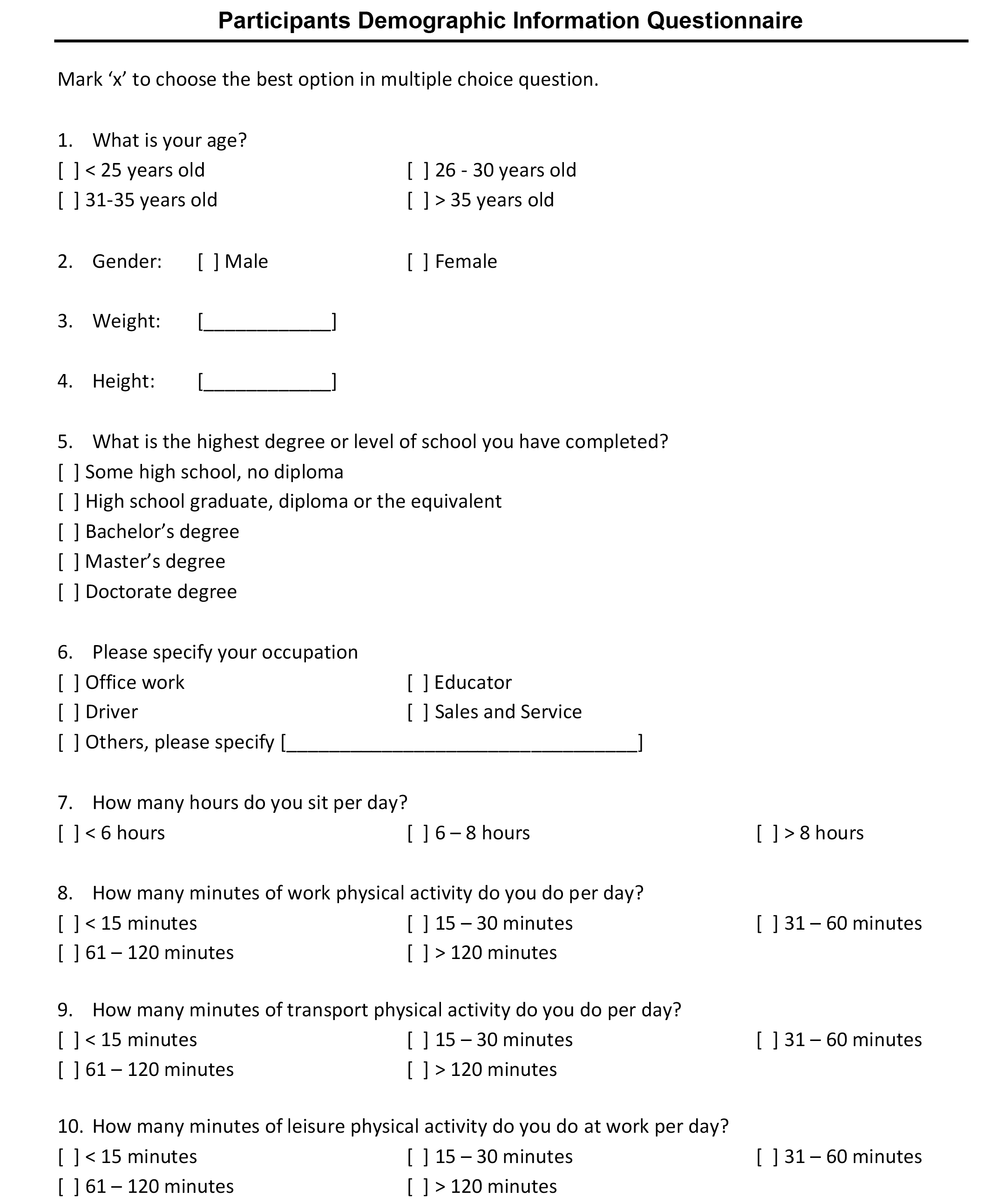

Supplement: Multimedia Appendix 1 [file formative_v6i1e34309_app1.png]

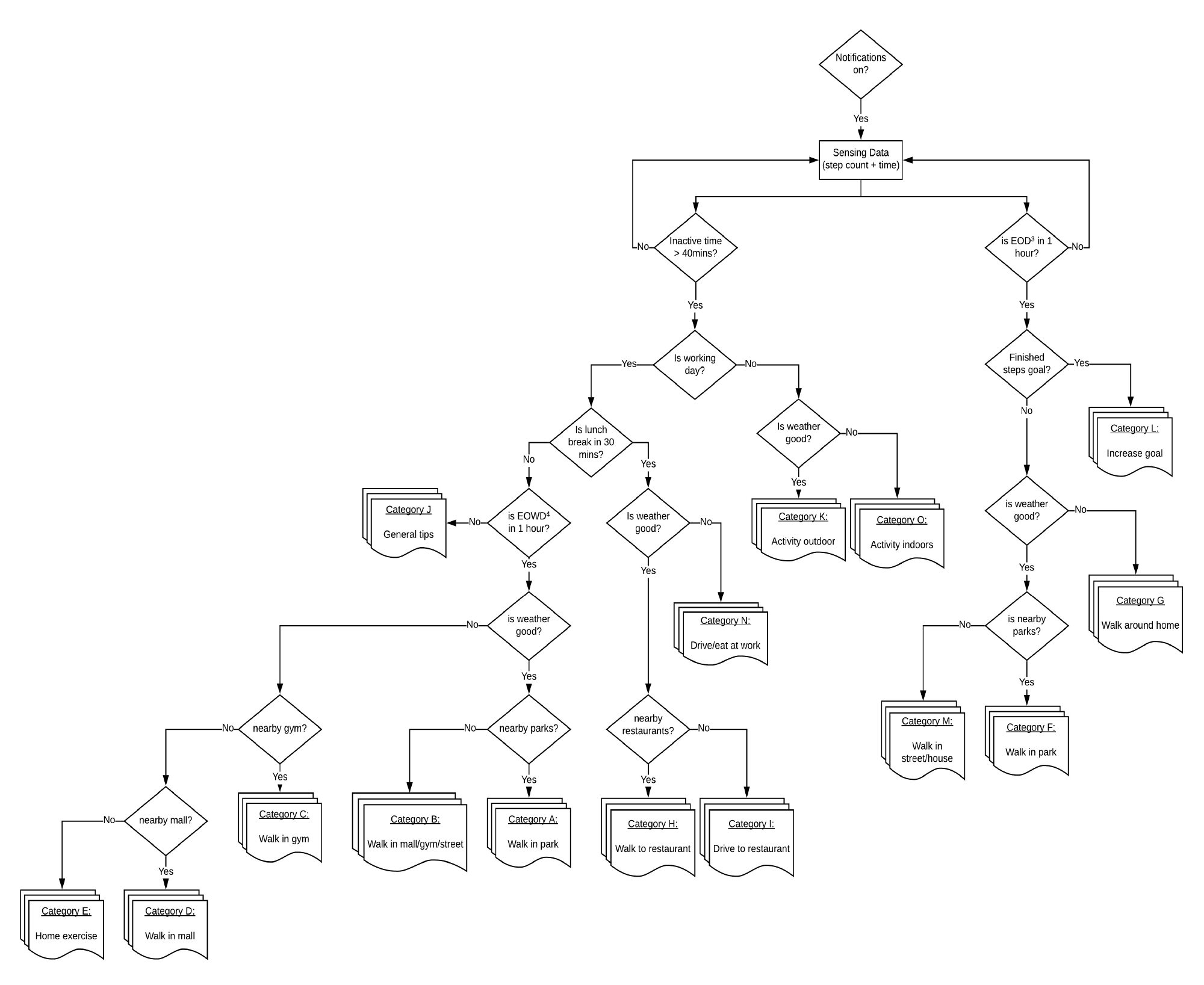

Supplement: Multimedia Appendix 3 [file formative_v6i1e34309_app3.png]
