# Supplementary material for: Design and Evaluation of a Just-in-Time Adaptive Intervention (JITAI) to Reduce Sedentary Behavior at Work: Experimental Study
Source: JMIR Form Res. 2022 Jan 26;6(1):e34309. doi: 10.2196/34309 (PMC8943689; doi:10.2196/34309)
Supplement: Multimedia Appendix 2 [file formative_v6i1e34309_app2.docx]

# Multimedia Appendix 2

Table 2 Dynamic context attributes in motivational messages

| Dynamic attributes | Description |
| --- | --- |
| <percentage-finished> | The percentage of the steps taken during the day compared to the steps goal |
| <percentage-remaining> | The percentage of the steps remaining until the daily steps goal is achieved compared the steps goal |
| <steps-remaining> | The number of steps remaining until the daily steps goal is achieved |
| <steps-goal> | The daily steps goal set up by the user |
| <weather> | The current temperature in degree Celsius (°C) |
| <name> | The username |
| <restaurants> | The name of a nearby restaurant |
| <park> | The name of a nearby park |
| <gym> | The name of a nearby gym |
| <mall> | The name of a nearby mall |
